# A trial-based cost-effectiveness analysis of antibiotic prescription strategies for non-complicated respiratory tract infections in children

**DOI:** 10.1186/s12887-023-04235-3

**Published:** 2023-10-02

**Authors:** Gemma Mas-Dalmau, María José Pérez-Lacasta, Pablo Alonso-Coello, Pedro Gorrotxategi-Gorrotxategi, Emma Argüelles-Prendes, Oscar Espinazo-Ramos, Teresa Valls-Duran, María Encarnación Gonzalo-Alonso, María Pilar Cortés-Viana, Tatiana Menéndez-Bada, Marta Esther Vázquez-Fernández, Ana Isabel Pérez-Hernández, Laura Muñoz-Ortiz, Carmen Villanueva-López, Paul Little, Mariam de la Poza-Abad, Misericòrdia Carles-Lavila, on behalf of the DAP Paediatrics Group, Josefa Manuel-Enguidanos, Natividad Herrero-Torres, Lorena Martínez-Villamizar, Carme Palassí-Bargalló, Maria Amor Peix-Galito, Francesca Camps-Serra, Rosa Mené-Bergara, Paloma Ramírez-Álvarez, Marisa Pietrafesa-Barreiro, Pilar Ortiz-Ros, Virgina del Rey-Márquez, Lucía Barahona-Rondón, María Rosario Benítez-Rubio, Ana María Valero-Marugán, María Laura Casado-Sánchez, Ángeles de Pando-Bravo, Miren Arrate Bengoa-Gorosabe, Carmen Callén-Blecua, Inés Hernández-Salvador, Irene Ozcoidi-Erro, Javier Eduardo Blanco-González, Carmelo Gutiérrez-Abad

**Affiliations:** 1 Department of Epidemiology and Public Health - Iberoamerican Cochrane Centre, Hospital de la Santa Creu i Sant Pau - Biomedical Research Institute Sant Pau (IIB Sant Pau), Barcelona, Spain; 2 Nursing Care Research Group, IIB Sant Pau, Barcelona, Spain; 3 Department of Economics, Universitat Rovira i Virgili, Reus, Spain; 4 Economic Challenges for the Next Generation (ECO-NEXT: SGR2021-00729), Reus, Spain; 5 Research Centre On Economics and Sustainability (ECO-SOS), Reus, Spain; 6 CIBER Epidemiology and Public Health (CIBERESP), Madrid, Spain; 7 Pasai San Pedro Primary Care Centre, Pasaia, Spain; 8 Ribadesella Primary Care Centre, Ribadesella, Spain; 9 Las Matas Primary Care Centre, Las Rozas de Madrid, Spain; 10 Val Miñor Primary Care Centre, Nigrán, Spain; 11 Ugao Miraballes Primary Care Centre, Ugao Miraballes, Spain; 12 Arrigorriaga Primary Care Centre, Arrigorriaga, Spain; 13 Ariz–Basauri Primary Care Centre, Basauri, Spain; 14 Maragall Primary Care Centre, Barcelona, Spain; 15 Iruña de Oca Primary Care Centre, Nanclares de la Oca, Spain; 16 Arturo Eyries Primary Care Centre, Valladolid, Spain; 17 Torrelodones Primary Care Centre, Torrelodones, Spain; 18 Catalan Agency for Health Quality and Assessment (AQuAS), Barcelona, Spain; 19 Manso Primary Care Centre, Barcelona, Spain; 20 Aldermoor Primary Care Centre, Southampton, UK; 21 Dr Carles Ribas Primary Care Centre, Barcelona, Spain

**Keywords:** Cost effectiveness, Delayed antibiotic prescription, Respiratory tract infections, Primary care, Paediatrics

## Abstract

**Background:**

Antibiotic prescription for respiratory tract infections (RTIs) in children attending primary care centres is almost double that predicted according to bacterial prevalence. Delayed antibiotic prescription (DAP) is designed to deploy a more rational use of antibiotics. While studies have evaluated DAP efficacy and safety for children with RTIs, little research has been conducted on the economic implications.

**Methods:**

Our trial compared cost-effectiveness for DAP, immediate antibiotic prescription (IAP), and no antibiotic prescription (NAP) for children aged 2–14 years with acute uncomplicated RTIs attended to in 39 primary care centres in Spain. The main outcome was the incremental cost-effectiveness ratio (ICER), measured in euros per gained quality-adjusted life days (QALDs). Net monetary benefit (NMB) was also calculated as a tool for decision making. The analysis was performed from a societal perspective for a time horizon of 30 days, and included healthcare direct costs, non-healthcare direct and indirect costs, and the antimicrobial resistance (AMR) cost.

**Results:**

DAP was the most cost-effective strategy, even when the cost of AMR was included. QALD values for the three strategies were very similar. IAP compared to DAP was more costly (109.68 vs 100.90 euros) and similarly effective (27.88 vs 27.94 QALDs). DAP compared to NAP was more costly (100.90 vs 97.48 euros) and more effective (27.94 vs. 27.82 QALDs). The ICER for DAP compared to NAP was 28.84 euros per QALD. The deterministic sensitivity analysis indicated that non-healthcare indirect costs had the greatest impact on the ICER. The cost-effectiveness acceptability curve showed that DAP was the preferred option in approximately 81.75% of Monte Carlo iterations, assuming a willingness-to-pay value of 82.2 euros per gained QALD.

**Conclusions:**

When clinicians are in doubt about whether an antibiotic is needed for children with RTIs attending PC centres, those treated with the DAP strategy will have slightly better efficiency outcomes than those treated with IAP because its costs are lower than those of IAP. DAP is also the most cost-effective strategy over a time horizon of 30 days if AMR is considered, despite higher short-term costs than NAP. However, if in the long term the costs of AMR are larger than estimated, NAP could also be an alternative strategy.

**Trial registration:**

This trial has been registered at www.clinicaltrials.gov (identifier NCT01800747; Date: 28/02/2013 (retrospectively registered).

## Background

One of the most frequent reasons for antibiotic prescription to children in primary care (PC) is a respiratory tract infection (RTI), [1] representing a significant economic burden for the health system [2]. The rate of outpatient antibiotic prescription for RTIs in children is high, [3–5] at almost double the rate predicted according to bacterial prevalence [3]. Most RTIs have a viral aetiology and are self-limiting, but antibiotics are indicated if a bacterial infection is suspected. Antibiotic prescription is typically associated with cases of diagnostic uncertainty [6–8] but is also the outcome of other factors, such as patient pressure for antibiotic prescription [9, 10]. Antibiotic prescription increases belief in efficacy and the demand for new consultations, [11, 12] although antibiotics are the most frequent cause of adverse effects in children, e.g., gastrointestinal and skin problems [13].

Over the long term, overuse of antibiotics is associated with bacterial resistance, [14] and reducing this resistance is a major global public health challenge [15]. According to the European Centre for Disease Prevention and Control (ECDPC), antimicrobial resistance (AMR) is responsible for approximately 33 110 deaths and 874 541 disability-adjusted life-years in the European Union/European Economic Area (EU/EEA) [16]. While this impact is recognized, relatively few countries have specific actions in place to reduce antibiotic intake.

Delayed antibiotic prescription (DAP) for RTIs, a strategy designed to foster more rational use of antibiotics, is recommended by clinical practice guidelines when there is uncertainty regarding immediate antibiotic prescription (IAP) [17, 18]. DAP is defined as a prescription issued for an antibiotic to be taken only if the condition has not improved or has worsened some days after the visit. A recent individual-patient-data meta-analysis comparing DAP, IAP, and no antibiotic prescription (NAP) reported that RTI symptom severity was similar for DAP and IAP, symptom duration was around the same for DAP and NAP and slightly shorter for IAP, re-consultations and complication rates were lower for DAP versus NAP, and patient satisfaction was higher for DAP [19].

While several randomized clinical trials (RCTs) have evaluated the efficacy and safety of DAP in children with RTIs, [19] there is a lack of cost-effectiveness studies evaluating antibiotic prescription strategies [20–23] in paediatric populations. Studies that do exist have focused on otitis media and have been carried out in the USA [20, 21, 23] and Canada [22]. Those studies have one important limitation: the cost of AMR was not taken into account [20–23].

Although IAP, the current form of treatment, is slightly more effective than DAP, according to a recent meta-analysis [19], previous economic analyses also conclude that DAP is the least costly strategy [20, 21], because it implies less antibiotic consumption, and fewer adverse effects. Therefore, DAP will likely be more cost-effective than IAP. The differences between DAP and NAP, however, are not easy to determine, since the results may depend on the complications derived from the non-use of antibiotics, and/or on the adverse effects derived from antibiotic use. Finally, it should be noted that the impact of DAP in reducing AMR cannot be appreciated over the short term. For children with RTIs, therefore, our aim was to analyse the overall cost-effectiveness of the DAP, IAP, and NAP strategies, including, in addition, an estimate of the AMR cost. This study was conducted in the context of a RCT [24].

## Methods

### Design

Trial-based cost-effectiveness analysis.

### Randomized clinical trial

The RCT [24] compared three antibiotic treatment strategies (DAP, IAP, and NAP), deployed in children with acute uncomplicated RTIs. Recruitment took place between June 2012 and June 2016 in 39 centres in Spain. Participants were children aged 2–14 years who attended with one of the following conditions: pharyngitis, rhinosinusitis, acute bronchitis, or acute otitis media. Children were included if paediatricians had a reasonable doubt about the need to prescribe an antibiotic. Children with pharyngitis were excluded when paediatricians had access to rapid streptococcal testing.

Prescription strategies were as follows:

Immediate antibiotic prescription: an antibiotic was prescribed to be started immediately on the day of the visit.

Delayed antibiotic prescription: an antibiotic was prescribed, but not to be started immediately; rather, parents were given structured recommendations about when to administer the antibiotic and when to consider returning to the paediatrician.

No antibiotic prescription: no antibiotic was prescribed, but parents were given structured recommendations about when to consider returning to the paediatrician.

For both the DAP and IAP strategies, each paediatrician decided the type of antibiotic to prescribe.

Primary outcomes were symptom duration and symptom severity. Symptom duration was measured as days until symptoms disappeared. Symptom severity was collected by parents using a 7-point Likert scale (0 = absence of symptoms, 1–2 = mild symptoms, 3–4 = moderate symptoms, and 5–6 = severe symptoms). Secondary outcomes were antibiotic use, additional visits, complications at 30 days, and beliefs and satisfaction of the parents.

Data were collected by paediatricians at the initial visit. Follow-up data were collected by telephone on days 2 and 30 after inclusion, and additionally, on days 7,15 and 22 when parents stated in the previous telephone call that symptoms persisted.

### Cost-effectiveness decision model

**Fig. 1:**
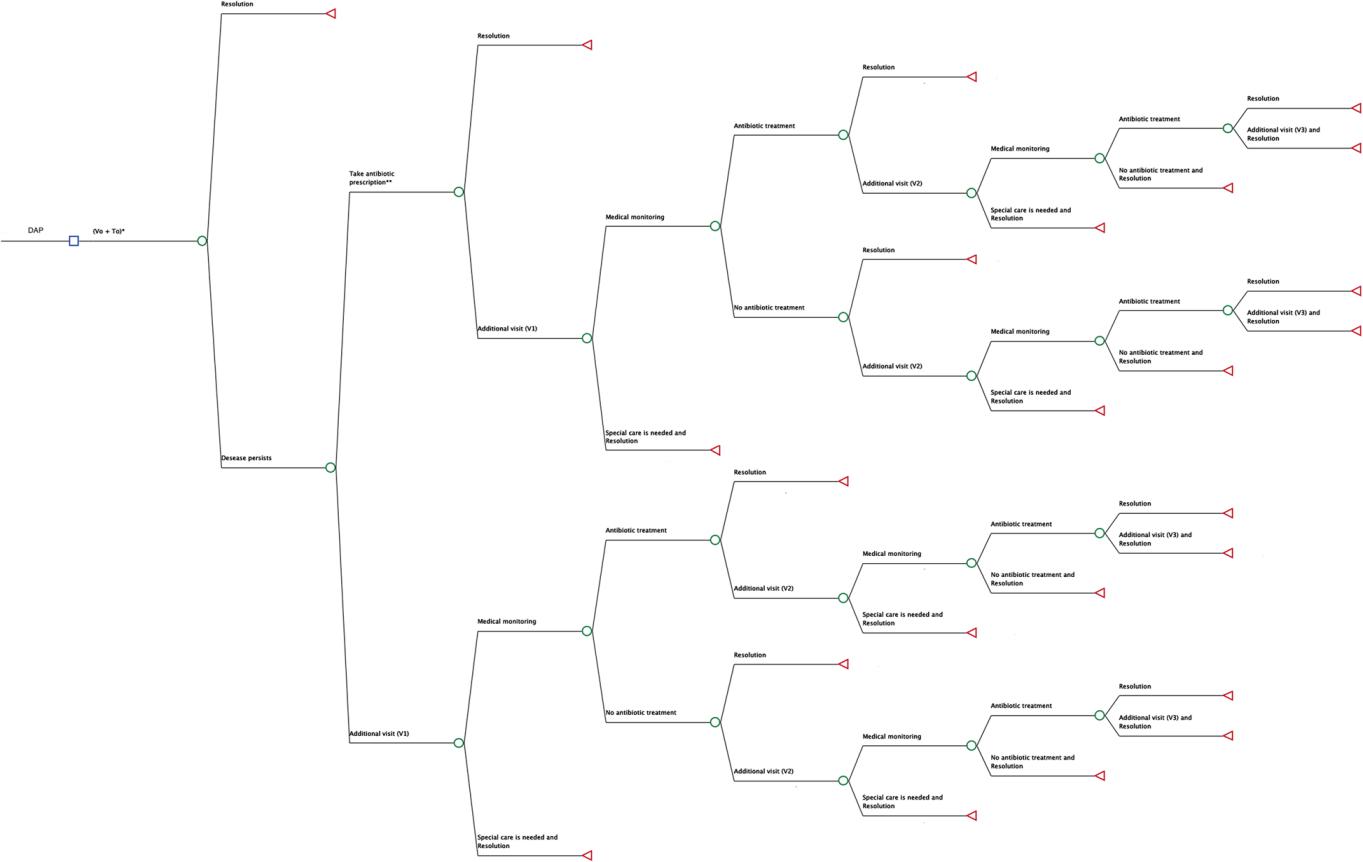
Decision tree. *V0 represents the baseline visit for the three strategies, each of which has a different initial treatment value (T0). DAP: delayed antibiotic prescription; IAP: antibiotic treatment; and NAP: no antibiotic. **If symptoms persist, the DAP alternatives are antibiotic prescription or a first additional primary care visit (V1). The only alternative for NAP and IAP is V1

A decision tree (Fig. 1) was created to compare the three strategies for a time frame of 30 days. A societal perspective was adopted that included healthcare direct costs, and non-healthcare direct and indirect costs. The three antibiotic strategies were deployed starting with a baseline visit (V0) in which, as the initial treatment, antibiotics were prescribed for the IAP and DAP arms, and no antibiotics were prescribed for the NAP arm. Two outcomes resulted following V0: (1) symptoms resolved; or (2) symptoms persisted. The response to those outcomes then depended on the original strategy assigned to each patient.

#### IAP and NAP

If symptoms persisted, the patient returned to the PC centre (V1). Two possible outcomes resulted following V1: (1) antibiotic treatment, either continuation (with the same or a different antibiotic) for the IAP arm, or prescription of an antibiotic to be started immediately for the NAP arm, or further waiting while continuing with the previous treatment; or (2) diagnosis and treatment of possible complications, specifically, pneumonia, abscesses, cellulitis, emergency department (ED) visits, and hospital admissions. The same procedure was applied to successive visits.

##### DAP

If symptoms failed to resolve after V0, parents could decide to either administer the prescribed antibiotic or return to the PC centre (V1). Following V1, the procedure was the same as for the IAP and NAP strategies.

The observed cases for each subtree and for each strategy, as represented in the decision tree (Fig. 1), were extracted from the RCT and are reported in Table 1.

**Table 1:** Model inputs: observed cases for each strategy

**Strategy**						**Cases (%)**
**DAP**	Resolution					70.71
	Disease persists	Take prescribed antibiotic	Resolution			20.00
			Additional visit (V1) / No antibiotic treatment / Resolution			1.43
		Additional visit (V1)	Antibiotic treatment / Resolution			3.57
			No antibiotic treatment	Resolution		2.14
				Additional visit (V2) / No antibiotic treatment / Resolution		1.43
			Special care is needed / Resolution			0.71
**IAP**	Resolution					91.61
	Disease persists, additional visit (V1)	No antibiotic treatment	Resolution			5,59
			Special care is needed / Resolution			0,70
			Additional visit (V2) / Antibiotic treatment		Resolution	0.70
					Special care is needed / Resolution	0.70
					Additional visit (V3) / No antibiotic treatment / Resolution	0.70
**NAP**	Resolution					87.77
	Disease persists, additional visit (V1)	Antibiotic treatment / Resolution				5.04
		Special care is needed / Resolution				1.44
		No antibiotic treatment	Resolution			5.04
			Additional visit (V2) / No antibiotic treatment / Resolution			0.72

*DAP* delayed antibiotic prescription, *IAP* immediate antibiotic prescription, *NAP* no antibiotic prescription

### Resource use and costs

Total costs, in euros for the year 2022, were calculated using a bottom-up costing approach (Table 2). Measurement data were collected during the RCT.

**Table 2:** Healthcare and non-healthcare costs

**Cost category**	**Measure**	**Data source**	**€ (2022)**	**Tornado diagram**	
				Variable	
				Low	High
**Healthcare direct costs**					
** Antibiotic medication**	mean standard treatment cost	Official prices [25]	5.20	4.42	5.98
Amoxicillin			4.58		
Amoxicillin-clavulanate			6.24		
Phenoxymethylpenicillin (penicillin V)			5.89		
Cefuroxime			11.62		
** Non-antibiotic medication**	mean standard treatment cost	Official prices [25]	2.50	2.13	2.88
** ED visits**	complications (minor emergency)	Rates 2020 [26]	215	182.75	247.25
	complications (non-urgent)	Rates 2020 [26]	130	110.50	149.50
** PC visits**	initial, additional, and adverse effects visits	Rates 2020 [26]	50	42.50	57.50
** Doctor time (NAP and DAP)**	mean 1 min	Research team consensus	5	4.25	5.75
**Non-healthcare direct and indirect costs**					
** Expenditure per visit**	no. of visits x (travel/visit time + transport + parking)		16.50	14.03	18.98
Time per visit	mean 40 min (travel/visit)	Research team consensus	10.50		
Transport per km	mean 0.20 €	Captio report [27]	1		
Parking per visit	mean cost	Rates 2022 [28]	5		
** Time lost to work**	hourly wage	INE [29]	15.85	13.47	18.23
**AMR cost**					
** AMR**	expected antibiotic cost, 30 days	Oppong et al. [30] Holmes et al. [31]	0.20	0.17	0.23

*AMR* antimicrobial resistance, *DAP* delayed antibiotic prescription, *ED* emergency department, *NAP* no antibiotic prescription, *PC* primary care

#### Healthcare direct costs

Costed were PC visits and ED visits, antibiotic and non-antibiotic medication use during the 30-day follow-up, doctor time (to explain recommendations for the assigned antibiotic strategy), and additional visits and drugs for adverse effects and complications (ED visits and hospital admissions). ED visits were evaluated by level of urgency as either non-urgent or a minor emergency. PC and ED visits were costed using data sourced from the Department of Health (Generalitat de Catalunya) [26]. Antibiotic and non-antibiotic medication costs were based on Spanish official prices. Considered were several classes of drugs currently in use: amoxicillin, amoxicillin-clavulanate, phenoxymethylpenicillin, and cefuroxime as antibiotics, and paracetamol and ibuprofen as non-antibiotic medication. Antibiotic and non-antibiotic medication costs were calculated from the number of packages needed to dose a 6-year-old child (the mean age of the included children). Doctor time to explain DAP and NAP strategy recommendations was calculated as equivalent to an additional 10% of the standard consultation time (i.e., 1 min per mean 10-min visit). No doctor time was counted for IAP as this strategy was considered the usual option.

In relation to adverse effects, we assumed that a rate of 10% in children treated with antibiotics, similar to the rate reported in the analysis by Coco et al. [20] and reflecting our RCT. We also assumed that adverse effects would always involve an additional visit and sometimes the prescription of non-antibiotic medication.

#### Non-healthcare direct and indirect costs

Direct costs calculated, using secondary information sources, were travel to healthcare institutions, parking, and outpatient consultation time for parents, while an indirect cost was time lost to work by parents (measured using a human capital approach), calculated from hourly wage data obtained from the Spanish National Statistics Institute (INE) [29]. Data on time lost to work were collected during the RCT. This cost was included, irrespective of who finally assumed it (the employer or the individual).

#### AMR cost

AMR was costed per prescription and per day using data published by the ECDPC [32] and the methodology of Oppong et al. [30]. Assuming that the Spanish population represents approximately 9% of the EU/EEA population and that prescriptions are made for seven days, we estimated 0.20 euros (2022) as the AMR cost per prescription over 30 days. The European average is similar to this value (0.15 pounds sterling, equivalent to 0.18 euros) according to Holmes et al. [31].

While AMR was included in our model as a cost, the cost of the reduction in antibiotic effectiveness assumed by society was not included, as is the usual practice in economic evaluations, due to the complexity of calculating this cost [30].

All costs were included in a deterministic sensitivity analysis (tornado diagram) whose low–high range is shown in Table 2.

### Effectiveness estimates

Effectiveness estimates were calculated from quality-adjusted life-days (QALDs). QALD was used rather than quality-adjusted life year (QALY) because our time horizon was 30 days [20]. QALDs were calculated by multiplying days in each health state (moderate or severe specific symptoms, adverse effects, and days without symptoms), as collected during the RCT, by the associated utility value reflecting the child’s health-related quality of life at a given point in time.

Utility is normally scaled from 0 (= death) to 1 (= perfect health) and utility values for different health states in children with non-complicated RTIs are reported in the literature [20, 21, 23]. However, since, in our RCT, parents reported their children’s health state using a visual analogue scale (VAS), scored from 0 (= worst state) to 100 (= best state), the utility values used were based on those VAS scores.

The QALD value for a 30-day period for a child in perfect health is 30. Days of main moderate or severe specific symptoms and adverse effects indicated disutility, which we calculated as the difference between 1 and the average VAS score for each strategy. Data were collected to calculate utility as follows: on day 2, in relation to main severe specific symptoms, on day 7 in relation to main moderate specific symptoms, and on day 30 for no specific symptoms. Those days were chosen based on the mean duration in days for the main severe specific symptoms of 2.4 for DAP, 2.6 for IAP, and 2.6 for NAP, and for main moderate specific symptoms of 7 for DAP, 6.9 for IAP, and 6.9 for NAP.

For adverse effects, we calculated the number of days of adverse effects according to Coco et al. [20], and the associated disutility as reported by Shaikn et al. [21]. The disutility value for gastrointestinal adverse effects was 0.12, assuming that diarrhoea is a common, 2-day adverse effect, in 10% of children that used antibiotics [21]. In the case of hospitalization, disutility was rated as equivalent to the main severe symptoms and main moderate symptoms by consensus of the research team. Utility values and average days in each health state are reported in Table 3. A probabilistic sensitivity analysis was performed for these values.

**Table 3:** Health state: utilities and average days

Health state	**Utility value (days on average)**			Reference
	**DAP**	**IAP**	**NAP**	
Zero symptoms	0.969 (20.57)	0.96 (20.50)	0.963 (20.58)	Mas-Dalmau et al. [24]
Severe symptoms	0.776 (2.39)	0.782 (2.57)	0.773 (2.57)	Mas-Dalmau et al. [24]
Moderate symptoms	0.875 (7.04)	0.897 (6.94)	0.879 (6.86)	Mas-Dalmau et al. [24]
Adverse effect disutility	0.12 (0.05)	0.12 (0.20)	0.12 (0.02)	Shaikn et al. [21] / Coco [20]

*DAP* delayed antibiotic prescription, *IAP* immediate antibiotic prescription, *NAP* no antibiotic prescription

### Analyses

The three arms of the decision tree were compared in terms of cost per QALD using the ICER for the non-dominated alternatives. Cost-effectiveness analysis results were generated by summing direct health costs, non-healthcare direct and indirect costs, and AMR cost in euros per patient treated to obtain a total cost. Effectiveness was measured in gained QALDs, also per patient treated. The options, presented in order of costs (lowest to highest) were assumed to be mutually exclusive (a patient can only receive one intervention at a time). Dominated alternatives were excluded. Of the two dominance types, strict and extended, an alternative had strict dominance if it was less costly and yet more effective, and had extended dominance if its ICER was greater than the ICER of the next most effective alternative. We also calculated the net monetary benefit (NMB) [33] was also calculated as a better tool for decision making. Since the time horizon of the model was short (30 days), no discount rate over time was calculated.

We conducted a deterministic sensitivity analysis for all costs as listed in Table 2. The tornado analysis tested multi-way effects on the results of the model, reflecting the impact of variations in the ICER, which oscillated between low and high in a range from minus 15% to plus 15%.

We also conducted a probabilistic sensitivity analysis, in which all parameters were simultaneously and randomly varied across 10 000 Monte Carlo iterations in order to calculate cost-effectiveness probabilities for the three strategies. Distributions used were a beta distribution for utilities and probabilities, and a gamma distribution for costs [33].

An incremental cost-effectiveness plane and a cost-effectiveness acceptability curve were plotted to calculate the probability that an alternative may be cost-effective, given a threshold range of values (0–164.4 euros) for willingness-to-pay. The willingness-to-pay value was defined as the maximum cost that a society is willing to pay for one QALD gained in health. We considered a willingness-to-pay value of 82.2 euros per day, in accordance with the recommended 30 000 euros/QALY [21, 29].

TreeAge Pro 2021 (TreeAge, Williamstown, Massachusetts) statistical software was used for the analyses.

## Results

### Patients and clinical outcomes

A total of 422 paediatric patients were included in the trial. Mean (SD) age was 6.3 (3.0) years, and 216 (51.2%) were girls. Diagnoses were acute otitis media (*n* = 217; 51.4%), pharyngitis (*n* = 141; 33.4%), bronchitis (*n* = 39; 9.2%), and rhinosinusitis (*n* = 25, 5.9%). Most children (*n* = 382; 90.5%) had no respiratory comorbidities. Table 4 summarizes patient sociodemographic and clinical characteristics.

**Table 4:** Patient sociodemographic and clinical characteristics

	Measure	IAP	DAP	NAP	Total
		(*n* = 143)	(*n* = 140)	(*n* = 139)	(*n* = 422)
Age	mean (SD), years	6.4 (3.1)	6.4 (3.2)	6.1 (2.8)	6.3 (3.0)
Girls	cases (%)	75 (52.5)	64 (45.7)	77 (55.4)	216 (51.2)
Respiratory comorbidity	number (%)	15 (10.5)	14 (10.0)	11 (7.9)	40 (9.5)
Respiratory infection	cases (%)				
Rhinosinusitis		8 (5.6)	9 (6.4)	8 (5.8)	25 (5.9)
Pharyngitis		46 (32.2)	46 (32.9)	49 (35.3)	141 (33.4)
Acute bronchitis		14 (9.8)	12 (8.6)	13 (9.4)	39 (9.2)
Acute otitis media		75 (52.5)	73 (52.1)	69 (49.6)	217 (51.4)
Main specific symptoms^a^	mean (SD)				
Severe (5–6)	days	2.6 (5.4)	2.4 (6)	2.6 (6.5)	3 (6)
Moderate (3–4)		6.9 (5.8)	7 (6.3)	6.9 (7.9)	6.95(6.7)
Complications^b^	cases	2	1	2	5
Antibiotics^c^	prescriptions used	146	35	17	198
Non-antibiotic medication^c^	prescriptions used	173	200	231	604
Additional PC visits^c^	visits, number	16	15	18	49

Data are reported as frequencies and percentages except otherwise indicated

*DAP* delayed antibiotic prescription, *IAP* immediate antibiotic prescription, *NAP*: no antibiotic prescription

^a^Fever or difficulty swallowing for pharyngitis, earache for acute otitis media, breathlessness for rhinosinusitis, and breathlessness or chest breathing noise for acute bronchitis

^b^Perforated eardrum (*n* = 1); hospitalization for dehydration (*n* = 1); ED visits (*n* = 3)

^c^Data are reported in terms of frequency, as a patient may have used more than one antibiotic or non-antibiotic drugs or may have visited more than once

Note that of five ED visits, three were non-urgent and two were minor emergencies. A single DAP case of hospitalization for dehydration due to fever was considered an outlier because of its undue impact on the overall results (given its very high cost), and also because it had the same probability of occurring in any of three arms and mainly depended on risk factors such as the age of child. This case was therefore costed as a minor emergency.

### Cost-effectiveness

The cost-effectiveness of each antibiotic strategy is shown in Table 5, ordered from least to most costly. The ICER calculation allowed us to determine which strategies were dominated or excluded.

**Table 5:** Cost-effectiveness ranking

	**Strategy**	**Cost** (euros, 2022)	**Incremental cost** (euros)	**Effectiveness** (QALDs)	**Incremental effectiveness** (QALDs)	**ICER (**euros/QALDs)	**NMB**
**Category** **(excluding dominated)**	NAP	97.48		27.82			2189.58
	DAP	100.90	3.42	27.94	0.12	28.84	2195.91
**Category (all)**	NAP	97.48		27.82			2189.58
	DAP	100.90	3.42	27.94	0.12	28.84	2195.91
	IAP	109.68	8.78	27.88	-0.06	-148.11	2182.26

*DAP* delayed antibiotic prescription, *IAP* immediate antibiotic prescription, *ICER* incremental cost-effectiveness ratio, *NAP* no antibiotic prescription, *NMB* net monetary benefit, *QALD* quality-adjusted life days

Total costs in euros were 109.68 for IAP, 100.90 for DAP, and 97.48 for NAP. QALDs were 27.94 for DAP, 27.88 for IAP, and 27.82 for NAP. DAP was the most cost-effective strategy overall. IAP was more costly but equally as effective as DAP. NAP was both less costly and less effective than DAP (by 0.12 QALDs). Comparing DAP with NAP, the ICER was 28.84 euros per gained QALD for DAP. The NMB results confirmed that DAP should be the preferred strategy, although the difference between DAP and NAP was very small (6.33 euros). The very similar QALD results for the three strategies approximate our analysis to a cost minimization analysis (Table 5).

### Deterministic sensitivity analysis

**Fig. 2:**
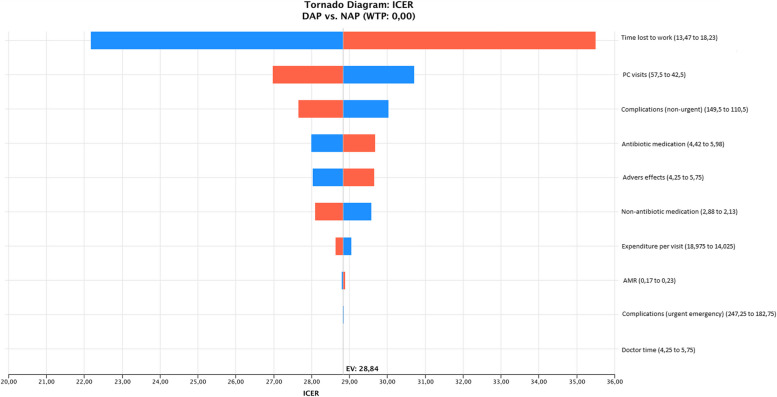
Deterministic sensitivity analysis: tornado diagram. ICER: incremental cost-effectiveness ratio. DAP: delayed antibiotic prescription; NAP: no antibiotic prescription

The cost of parental time lost to work, followed by the cost of PC visits, were the variables with the greatest impact on the ICER (Fig. 2). The relationship between time lost to work and impact on ICER was positive (in red to the right of the ICER value), while the relationship between PC visits and impact on ICER was negative (in blue to the right of the ICER value). Thus, any increase in time lost to work by parents increased the ICER value, while any increase in PC visits reduced the ICER value. This is explained by the fact that time lost to work affected DAP more than NAP, while PC visits affected NAP more than DAP.

The impact on the ICER was greater than the variation in the baseline value only in the case of time lost to work, with a variation of 15% in time lost to work having a 23% impact on the ICER **(**Table 6**)**.

**Table 6:** Tornado results

**Variable**	**Impact**	**Low ICER**	**High ICER**	**Cum Risk %**
Time lost to work	Increase	22.18	35.50	0.87
PC visits	Decrease	26.97	30.71	0.93
Complications (non-urgent)	Decrease	27.66	30.02	0.96
Antibiotic medication	Increase	28.00	29.68	0.98
Adverse effects	Increase	28.03	29.65	0.99
Non-antibiotic medication	Decrease	28.09	29.57	1.00
Expenditure per visit	Decrease	28.64	29.04	1.00
AMR	Increase	28.81	28.87	1.00
Complications (minor emergency)	Decrease	28.83	28.85	1.00
Doctor time (for DAP and NAP)	Increase	28.84	28.84	1.00

*AMR* antimicrobial resistance, *DAP* delayed antibiotic prescription, *ICER* incremental cost-effectiveness ratio, *NAP* no antibiotic prescription, *PC* primary care

### Probabilistic sensitivity analysis

**Fig. 3:**
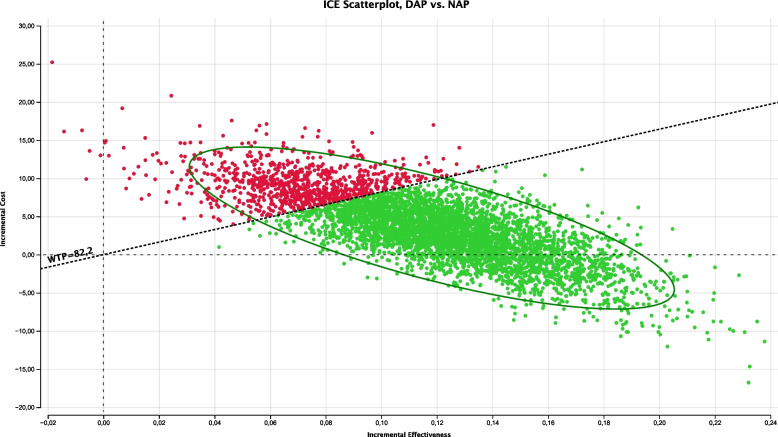
Probabilistic sensitivity analysis: cost-effectiveness plane. DAP: delayed antibiotic prescription; NAP: no antibiotic prescription; WTP: willingness-to-pay

Figure 3 shows the incremental cost-effectiveness scatterplot with 10 000 Monte Carlo iterations of the probabilistic model. DAP was more effective and more costly than NAP, as indicated by the 79.10% of iterations in quadrant I; however, the fact that ICER was below the willingness-to-pay value (82.2 euros) for 61.34% of the iterations in quadrant I indicates that DAP was the societally eligible strategy. Furthermore, DAP could be a dominated option in 20.77% of the iterations (represented in quadrant IV).

**Fig. 4:**
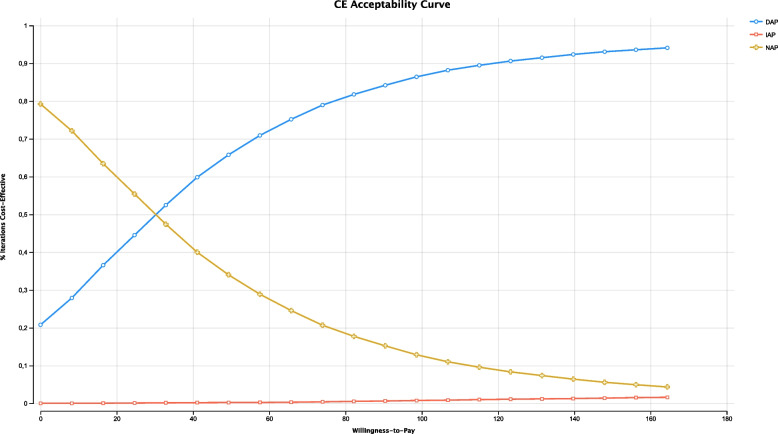
Probabilistic sensitivity analysis: cost-effectiveness acceptability curve. DAP: delayed antibiotic prescription; IAP: immediate antibiotic prescription: NAP: no antibiotic prescription

Figure 4 depicts the cost-effectiveness acceptability curve for the probabilistic sensitivity analysis. As the willingness-to-pay value increased, DAP became more eligible, i.e., the probability of cost-effective iterations increased for the DAP strategy. For a willingness-to-pay value of 82.2 euros, DAP compared to NAP accounted for around 81.75% of cost-effectiveness iterations. For any willingness-to-pay value, IAP was dominated by one or both of the other alternatives.

## Discussion

### Main findings

DAP was the most cost-effective strategy for children aged 2–14 years attending PC centres with RTIs, whose paediatricians had reasonable doubt about the need to prescribe an antibiotic. NAP was less costly but less effective than DAP, although the difference was very small (0.12 QALDs). The ICER of DAP compared to NAP was 28.84 euros per gained QALD. The probabilistic sensitivity analysis showed that DAP was more cost-effective than NAP in 81.75% of the Monte Carlo iterations, with 82.2 euros as the willingness-to-pay value based on the recommended 30 000 euros/QALY. IAP was the dominated strategy, as it cost more and was equally as effective as DAP. The deterministic sensitivity analysis showed that time lost to work and PC visits were the costs with most impact on ICER values. Inclusion of the AMR cost in the analysis, referring to an interval of 30 days, did not change the results.

### Results in context

Two previous studies [20, 21] have evaluated the cost-effectiveness of different antibiotic prescription strategies, including DAP. IAP in our study was more costly than DAP, as in the studies by Coco et al. [20] and Shaikh et al. [21] both of which also adopted a societal perspective. However, in our study, the cost difference between IAP and DAP (8.78 euros) was less than the 22.9 US dollars (DAP compared to IAP with 7–10 days of amoxicillin) for the Coco et al. study, and 36.37 US dollars (DAP compared to IAP with amoxicillin) for the Shaikh et al. study. The incremental gain in QALDs between strategies was very small in our study, as was the case in the above-mentioned two studies. Nevertheless, in those studies, IAP was the most cost-effective strategy, whereas in our study, DAP was the most cost-effective strategy. Our incremental gain in QALDs for DAP compared to IAP was 1.44 h, compared to the incremental gain in QALDs for IAP compared to DAP of 8.6 h in the Shaikh et al. study (IAP with amoxicillin), and 3.5 h in the Coco et al. study (IAP with 7–10 days of amoxicillin). Once the lower use of antibiotics and the lower adverse effects that can occur in DAP are considered, a possible explanation for the differences could be that our disutility values for the health status of children randomized to DAP, based on parent-reported VAS values, were lower than in previous studies.

Two other studies have evaluated the cost-effectiveness of different antibiotic prescription strategies but without including the DAP option. Sun et al., [23] in a study which was also based on a societal perspective, applied a watchful waiting approach as recommended in American Academy of Pediatrics guidelines, i.e., an antibiotic is considered for prescription only after waiting to see if symptoms would self-resolve. On the basis that watchful waiting could be considered a similar strategy to DAP, our finding that DAP was the most cost-effective strategy corroborates that Sun et al. [23] finding that watchful waiting was the most cost-effective strategy. Gaboury et al. [22] evaluated the cost-effectiveness of different antibiotic prescription strategies, not including DAP; they reported a result that coincides with Coco et al. [20] and Shaikh et al. [21], namely, that IAP was more cost-effective than watchful waiting. However, in the Gaboury et al. study, and contrasting with our study and those by Coco et al. and Shaikh et al. (adopting a societal perspective), watchful waiting compared to IAP with amoxicillin cost 9.48 Canadian dollars more.

Accounting for the AMR cost, to some extent our results coincide with the Oppong et al. [30] study that evaluated the cost-effectiveness of amoxicillin compared to placebo for adults with lower-RTIs attending PC centres. That study found that the dominant strategy did not change when AMR cost was included, but only for European data, i.e., not for data from other regions. Amoxicillin was the dominant strategy for those European data, while DAP was the cost-effective strategy in our study. Note, however, that the Oppong et al. study did not adopt, as we did, a societal perspective.

However, in comparisons between our findings and those of the above-cited studies, similarities and differences must be interpreted with care, both because of the variety of methods used and because those studies were carried out in the USA or Canada with their different health system models and healthcare costs.

### Limitations and strengths

Our study has several possible limitations. First, we approximated health status utility using a VAS instead of measuring health status using standard gamble or time-trade off, or classifying health status using a questionnaire like the EuroQoL-5D [34]. Nevertheless, our findings can be considered reliable, as the QALDs were based on RCT data, and largely corroborate those of the meta-analysis by Oh [35]. Second, while the 30-day time horizon is sufficient for certain conditions, including RTIs, it is insufficient to assess the benefits of reduced antibiotic consumption in relation to reduced AMR. Third, we did not take into account private medical consultations, even though 12.6% of the Spanish population has private health insurance [36]. However, this limitation was likely to have had a similar impact on all three strategies. The trial was underpowered for two important cost drivers, namely, re-consultations and hospital admissions (included as complications), and wider individual-patient-data evidence [19] suggests that these are both higher with NAP compared to DAP; we therefore may have underestimated the cost-effectiveness of DAP. Fourth, the study was conducted in a pre-COVID-19 pandemic scenario, i.e., before the introduction of new rapid tests that could reduce diagnostic uncertainty. The cost of such tests were not considered but, as a fixed cost, it would not modify the results.

Our study also has some strengths. The main ones are that the study was based on a pragmatic RCT and, in analysing the cost-effectiveness of different antibiotic prescription strategies for children with RTIs, is the first such study performed outside North America. Our study, based on previous literature and a time horizon of 30 days, also considers AMR cost, a key issue not included in previous studies [20–23]. Finally, included also was the impact of non-healthcare direct and indirect costs in our study, reflecting a societal perspective.

### Implications for practice and research

DAP is the most cost-effective strategy, although the difference with NAP is very small and the alternative IAP is a dominated strategy. For this reason, when panels consider the reduction of AMR a critical outcome, guideline panels are likely to recommend DAP strategies in those cases in which clinicians have doubts about whether it is necessary to administer an antibiotic to children with RTI.

Future studies should focus on more accurate analyses of the cost of AMR over a longer time period, and should consider the consequences of taking antibiotics not only in terms of costs, but also in terms of disutility of different health states, including re-consultations and complications.

## Conclusions

When clinicians are in doubt about whether an antibiotic is needed for children with RTIs attending PC centres, those treated with the DAP strategy will have slightly better efficiency outcomes than those treated with IAP because its costs are lower than those of IAP. DAP is also the most cost-effective strategy over a time horizon of 30 days if AMR is considered, despite higher short-term costs than NAP. However, if in the long term the costs of AMR are larger than estimated, NAP could also be an alternative strategy.

## Data Availability

Study data and materials not included in this article are available from the corresponding authors on reasonable request.

## Abbreviations

AMR: Antimicrobial resistance
CREC: Clinical research ethics committee
DAP: Delayed antibiotic prescription
ED: Emergency department
IAP: Immediate antibiotic prescription
ICER: Incremental cost-effectiveness ratio
NAP: No antibiotic prescription
NMB: Net monetary benefit
PC: Primary care
QALD: Quality-adjusted life day
QALY: Quality-adjusted life year
RCT: Randomized clinical trial
RTI: Respiratory tract infection
VAS: Visual analogue scale
